# Investigating the Usability of a Head-Mounted Display Augmented Reality Device in Elementary School Children

**DOI:** 10.3390/s21196623

**Published:** 2021-10-05

**Authors:** Luisa Lauer, Kristin Altmeyer, Sarah Malone, Michael Barz, Roland Brünken, Daniel Sonntag, Markus Peschel

**Affiliations:** 1 Department of Physics, Campus C6.3, Saarland University, 66123 Saarbrücken, Germany; markus.peschel@uni-saarland.de; 2 Department of Education, Campus A4.2, Saarland University, 66123 Saarbrücken, Germany; kristin.altmeyer@uni-saarland.de (K.A.); s.malone@mx.uni-saarland.de (S.M.);; 3 German Research Center for Artificial Intelligence (DFKI), Interactive Machine Learning Department, Stuhlsatzenhausweg 3, Saarland Informatics Campus D3_2, 66123 Saarbrücken, Germany; michael.barz@dfki.de (M.B.); daniel.sonntag@dfki.de (D.S.); 4 Applied Artificial Intelligence, Oldenburg University, Marie-Curie-Str. 1, 26129 Oldenburg, Germany

**Keywords:** head-mounted displays, augmented reality, human activity recognition, usability, elementary education

## Abstract

Augmenting reality via head-mounted displays (HMD-AR) is an emerging technology in education. The interactivity provided by HMD-AR devices is particularly promising for learning, but presents a challenge to human activity recognition, especially with children. Recent technological advances regarding speech and gesture recognition concerning Microsoft’s HoloLens 2 may address this prevailing issue. In a within-subjects study with 47 elementary school children (2nd to 6th grade), we examined the usability of the HoloLens 2 using a standardized tutorial on multimodal interaction in AR. The overall system usability was rated “good”. However, several behavioral metrics indicated that specific interaction modes differed in their efficiency. The results are of major importance for the development of learning applications in HMD-AR as they partially deviate from previous findings. In particular, the well-functioning recognition of children’s voice commands that we observed represents a novelty. Furthermore, we found different interaction preferences in HMD-AR among the children. We also found the use of HMD-AR to have a positive effect on children’s activity-related achievement emotions. Overall, our findings can serve as a basis for determining general requirements, possibilities, and limitations of the implementation of educational HMD-AR environments in elementary school classrooms.

## 1. Introduction

Augmented reality (AR) is an emerging technology in education that enables real-time integration of real and virtual objects in the field of view [1,2]. The real world represents the main channel of perception, and virtual objects are spatially and/or semantically connected to real objects [3]. In educational settings in particular, this offers a great potential to enhance learning processes and, therefore, there is a high interest in the development and research of AR-environments and devices in this context. In particular, head-mounted displays enable an engaging interaction with real and virtual objects. Recent review studies and meta-analyses have confirmed the general benefits of AR-applications for learning [4,5,6]. However, it is noticeable that children of elementary school age benefit less than older students [7]. The usability of the applied devices seems to play a significant role in the success of AR-applications [8]. Technology for the recognition of user activities and behavior is referred to as ‘human activity recognition’ (HAR) technology [9,10]. It is suspected that HAR of AR-devices such as Microsoft’s HoloLens (first generation) was not yet technologically mature enough to enable interference-free learning in younger children. However, AR-technology has evolved and may be able to make up for the shortcomings of the past, allowing the potential for AR to be suitable for younger students. Therefore, the current study aimed to test the usability of the HoloLens 2 for elementary school students and to provide an empirical basis to decide whether it is worthwhile to develop school-related learning scenarios for the device. In addition, we examined which of the offered multimodal interaction modes can be handled best by the students.

### 1.1. Theoretical and Empirical Background for the Use of HMD-AR in Education

Following Santos et al. [11], the overlay of the physical world with external representations through AR enables situated multimedia learning. Based on this approach, through AR it is attempted to combine the best of two worlds: situated active learning in a meaningful real-world environment and virtual learning environments carefully designed according to the principles of the Cognitive Theory of Multimedia Learning [12]. Initial studies indicate that through fulfilling the spatial contiguity principle, AR-based learning environments can reduce cognitive load [13,14] and increase learning gains [15]. Moreover, Szajna et al. [16] found that HMD-AR-based applications for training can significantly reduce the time required to perform tasks.

Most educational AR-applications are designed for handheld display-devices like smartphones or tablets, while head-mounted display AR-devices (HMD-AR-devices) are used rarely [4]. Nevertheless, HMD-AR-devices provide several advantages when used in educational settings. In contrast to handheld devices, learners wearing see-through HMD-AR-devices experience a seamless merge of virtual and physical worlds. From the perspective of multimedia learning, this should facilitate the creation of meaningful cognitive relations between virtual information and the physical environment, improving learning outcomes [12,17]. Moreover, unlike handheld display-devices, HMD-AR-devices allow for freehand interaction with physical as well as virtual objects [18]. This becomes particularly useful for learning settings based on physical activities, like laboratory work, which requires leaners to use both of their hands [19]. Theories of embodied cognition suggest that bodily interactions with a learning task, such as hand and finger gestures, can support cognitive processes [20]. Furthermore, Korbach et al. [21] used 2D multimedia learning material to show that using the index finger for pointing and tracing related information influences a learner’s focus of visual attention and promoted the learning process. Since HMD-AR-based learning environments enable the presentation or adaptation of learning information based on a learner’s gesture or action in real-time, positive effects of embodied cognitions are expected be particularly strong [22].

According to Yuen et al. [23], educational AR-applications can be designed for discovery-based learning (DBL), object modelling (OM), game-based learning (GBL), for the teaching of specific skills in training, or they can be integrated into distinct educational AR-books, with GBL and OM being the most frequently addressed purposes of educational AR-applications [24]. AR in education can help learners to conduct authentic explorations in the real world by displaying virtual elements [25], and can facilitate the observation of processes that cannot be perceived with the naked eye [26]. Further, AR opens new opportunities for the individualization of the learning process through real-time interaction between reality and virtuality as real-time reaction and adaption to the learner’s actions [27]. For instance, recent technological advances enable the augmentation of relevant real objects that were fixated by a learner [28]. Besides promoting the acquisition of knowledge and skills [5,29], AR can positively influence curiosity [30] as well as motivation and interest [31] in educational situations. Motivation and interest are known to be modulated by so-called activity emotions, which, as a type of achievement emotions, concern ongoing achievement-related activities [32,33]. While positive, activating emotions (e.g., enjoyment) are assumed to promote motivation and interest, negative, rather deactivating emotions (e.g., boredom), are associated with their decline. Therefore, emotions such as enjoyment, boredom, etc. are referred to as ‘activity-related achievement emotions’.

However, the use of AR in education can be obstructed by technical issues and can require additional instruction [34]. Hence, well-designed user interfaces in AR-applications are essential for successful learning [35]. Due to the prevailing research gap concerning the use of HMD-AR-devices and applications in education, as well as ongoing technical advancements concerning HMD-AR-devices, further research is required to validate the existing results and to investigate the effects of HMD-AR on the learning process.

### 1.2. Usability of HMD-AR-Devices in Education

In order for AR-devices to exert their positive impact on information processing during learning, the handling and interaction with the device itself or with the virtual learning information offered must not itself lead to load on the learners as described in the previous Section. According to several reviews on AR in education, an often-reported issue concerning the practical use of AR-devices and applications in educational situations is the underwhelming usability [4,36,37]. The (technical) usability of an educational technology-supported setting, which comprises technically conditioned aspects of use and operation, influences the overall usefulness of a learning application [38]. While good usability of educational AR-applications facilitates learning, poor usability can even hamper learning processes [39]. Further, Papakostas et al. [40] found the usability to be the strongest predictor of the behavioral intention to use an AR-application for training. For HMD-AR-devices, a poor performance of the user activity recognition concerning the detection of operation commands can impact usability, as the device is operated through gesture- or voice-based interaction [41]. This aspect is more important when using the devices with young children, as their physical body characteristics (e.g., hand size, arm length, voice pitch) differ from adults [42], for whom the devices are currently designed and calibrated. Previous research concerning the (technical) usability of HMD-AR-devices focused mainly on Microsoft’s HoloLens (first generation) and samples of adults. An evaluation of the device for the purpose of an assembly application for manufacturing [18] found the device to be applicable, but also revealed that the spatial mapping required improvement. Munsinger et al. [43] used the Microsoft HoloLens (first generation) to investigate its usability for a target group of elementary school children. They compared three AR-interaction modes provided by HoloLens (‘remote clicker’, ‘air-tap’, ‘voice command’) in their efficiency using the measures ‘input errors’ ‘tutorial time’ and ‘game time’, and found that the ‘voice command’-interaction performed significantly worse than the other two. Their findings are in line with rather poor performance occurring for interactive devices with voice-based operation in general [41,44]. Besides their physical body characteristics, the children’s individual state of cognitive development concerning motoric skills and spatial cognition [42] may affect the usability of HMD-AR-devices.

For many applications, multimodal interfaces have long been recognized to be more robust, accurate, and preferred by users than unimodal ones. A major benefit is that users can freely choose their preferred modality combination [45]. However, this requires the ability to make a good modality choice, because ineffective interaction modalities may lead to unsatisfactory results [46]. Still, multimodal interfaces are considered to be “especially well-suited for applications like education, which involve higher levels of load associated with mastering new content” [47] (p. 33). As the HoloLens 2 offers different means to multimodally interact in AR, we investigate the preferred modality choices of elementary school children. So far, investigations on children’s handling with the revised interaction modes of the latest HoloLens 2 are still pending.

### 1.3. The Microsoft HoloLens 2 and Its Potential for Education

**Figure 1:**
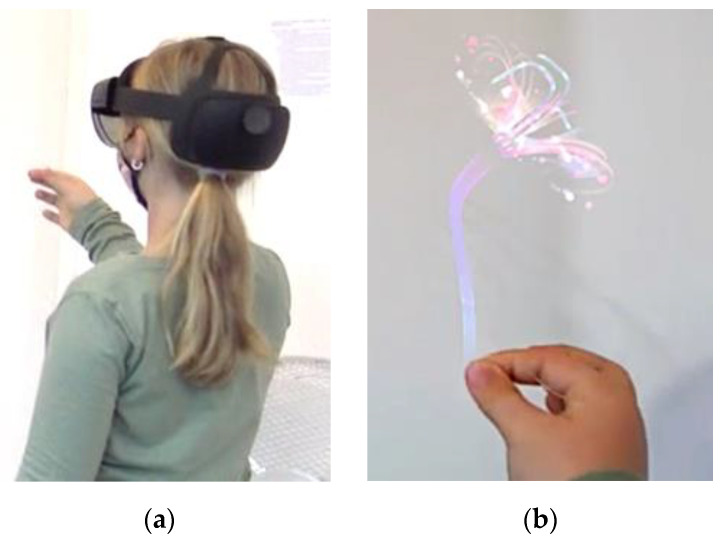
Child interacting with a virtual object in AR using Microsoft’s HoloLens 2. (**a**) external view; (**b**) child’s point of view.

Announced innovations and improvements concerning the spatial positioning, speech, and gesture recognition for the successor model HoloLens 2 by Microsoft (see Figure 1) do not only make it necessary to investigate the applicability of existing findings for the new device. The new device could further represent an important step towards user-friendly HMD-AR-applications for educational purposes, especially for young children.

**Figure 2:**
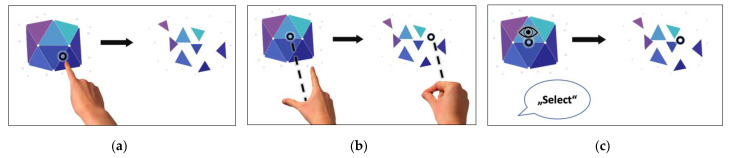
AR-interaction modes provided by HoloLens 2. (**a**) ‘tap’; (**b**) ‘air-tap’; (**c**) ‘voice command’.

The HoloLens 2 offers various means to interact in AR. To describe these modes of interactions, we will focus on the action ‘selection of an AR-object’ from the tutorial that is pre-installed on the device (see Video S1a). The AR-object to select in the tutorial was a shimmering gemstone. On the one hand, there are gesture-based interactions: To select the AR-gemstone with a gesture-based interaction, one can either tap directly on the gemstone (newly implemented ‘tap’-interaction, see Figure 2a and Video S1a) or one can aim at the gemstone from a distance with the open palm and then tap with the thumb and index finger (‘air-tap’-interaction, see Figure 2b and Video S1a). On the other hand, there is voice-and-gaze-based interaction: To select the AR-gemstone, one can also look at the gemstone and say ‘select’ (‘voice command’-interaction, see Figure 2c and Video S1a). In total, two gesture-based and one voice-and-gaze-based interaction mode are available for AR-interaction on the HoloLens 2.

### 1.4. Ethics of Using HMD-AR Devices in Elementary Education

Children are vulnerable and it is the responsibility of adults to protect them from possible harm. Technologies in research on children should therefore be applied very prudently. Particularly when using immersive technologies, such as AR and virtual reality (VR), special precautions should be taken [48]. To ensure that the psychological and cognitive state of the target group was taken into account, our research team included experts in the fields of infant mental development (psychologists) and elementary school pedagogy (teachers and researchers). These considerations led us to gently introduce the children to the technology: we first showed them the device and explained how it works in a child-friendly way. While they were using the smartglasses, an experimental supervisor was always on hand to help them. We also monitored the physical well-being of the children [49] by asking them repeatedly whether they experienced any discomfort in terms of simulator sickness. Moreover, the virtual content of the AR environment used does not contain frightening or startling elements. To protect the children’s data (e.g., eye movement recordings [50]), we used a private offline Wi-Fi to enable Mixed Reality Capture.

### 1.5. Aim of the Study

Our aim is to assess the usability of Microsoft’s HoloLens 2 as the latest HMD-AR-device for the use with elementary school children. The device is not yet technically designed for use with children younger than 13 years: young children’s lower interpupillary distance might hamper the perception of virtual objects [51]. Therefore, we want to explore how usable the device is in its current state, and which technical adaptions need to be carried out before the device can be successfully used with young children, following similar evaluations for the predecessor model by Munsinger et al. [43]. We want to gain an insight into the general challenges and benefits that can serve as baseline findings once the device is used in educational applications. Our main research focuses concerning the use of the device are:

1. Evaluation of the overall usability of the HoloLens 2 as an HMD-AR-device;
2. Comparison of the provided AR-interaction modes concerning their efficiency;
3. Assessment of the children’s interaction preference in HMD-AR;
4. Examination of the change in activity-related achievement emotions.

## 2. Materials and Methods

### 2.1. Sample

We invited 47 students (29% female, age: M = 9.3 years; SD = 0.9 years, 2nd to 6th grade) to participate in a laboratory study at the Saarland University. They took part in another study at the same day (either before or after attending this study), but the other study did not include the use of an HMD-AR-device. None of the children had previous experience with AR. In the beginning, we conducted test runs with four children (for procedure and instruction refinement, without data collection), so n = 43 valid data sets were collected.

### 2.2. Study Design

The study was conducted using a within-subjects design. The independent variable was interaction mode, and the modes were modeled as different measuring points. The different multimodal AR-interaction modes provided by HoloLens 2 (‘tap’, ‘air-tap’, and ‘voice command’) were compared regarding the dependent variables ‘mean number of attempts’ and ‘mean time’. For the children’s personal interaction preference in AR, we formed the variables ‘most favorite interaction mode’ and ‘least favorite interaction mode’. The most and the least favorite interaction mode can be ‘tap’, ‘air-tap’ or ‘voice command’. To investigate general effects of HMD-AR-usage on activity-related achievement emotions, we formed a pre- and a post-test variable for ‘enjoyment’, ‘boredom’ and ‘frustration’. To assess the overall device usability, we formed the variable ‘system usability score’.

### 2.3. Procedure and Data Collection

**Figure 3:**
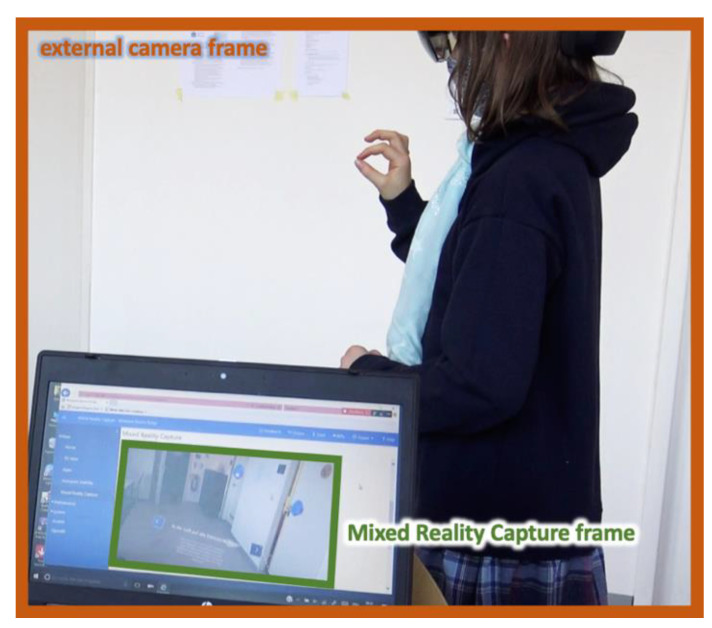
Video recording during the tutorial: An external camera and the ‘Mixed Reality Capture’ accessible through Microsoft Device Portal (via Browser) were used.

Due to the COVID-19 situation, only individual appointments with private journeys could be made. Prior to the start of the study, parents were informed about the investigation and gave their written consent for their children’s study participation. The procedure of the study was centered around a standardized tutorial on interaction in HMD-AR on the HoloLens 2 in German language. Before starting the tutorial, we assessed the children’s enjoyment, boredom and frustration. These activity-related achievement emotions are assumed to allow for inferences about motivation and interest [32,33] (variables ‘enjoyment-pre’, frustration-pre’, ‘boredom-pre’). Each emotion was assessed using a single item adapted for children from Riemer and Schrader [52] (see Appendix A and Document S1b). Moreover, children were asked about their previous experience with AR. The children were then introduced to the HoloLens 2 and the concept of HMD-AR by showing them the ‘Mixed Reality Capture’ (livestream) while the experimental supervisor was wearing the device (see Figure 3). We thoroughly instructed the children to handle the device carefully and explained that it is not a toy. Afterwards, the experimenter mounted the device on the child’s head and an eye calibration was carried out. As described in Section 1.5, the device is currently designed for adults and the manual states that children under the age of 13 years might not be able to see virtual objects comfortably due to a low interpupillary distance. We therefore asked the children after the eye calibration whether they had any problems in seeing the virtual objects, especially reading texts. Then, the children were informed that they were going to learn different methods of interaction in AR and went through the standardized tutorial on multimodal interaction in HMD-AR that is pre-installed on the HoloLens 2 (see Video S1a). The tutorial includes several interaction scenarios. For our analysis, we focus on the task ‘selecting a gemstone’ only because it is available for all interaction modes. The task ‘selecting a gemstone’ is the first shown in the tutorial. The three interaction modes are introduced one after the other and the order of the tutorial tasks is fixed (‘tap’—‘air-tap’—‘voice command’). At the beginning, three gems are shown. They must be selected one after the other with the respective method. During the entire tutorial, the gems can only be selected with the interaction method that is currently being introduced. An invisible speech-based virtual agent explains what to do in each case, and this information is additionally displayed in text form. For ‘tap’ the translated instruction is: “Tap a nearby gem with your finger to select it.” The translated instruction for ‘air-tap’ is: “Aim the beam from your palm at holograms out of range. Tap to select with your index finger and thumb and release.” For the ‘voice-command’-interaction, the translated instruction is: “Target a gem with the gaze cursor and say ‘Select’). The auditory explanation is played only once, while the text remains visible. If the correct (gesture or voice) input does not follow immediately, help is given depending on the interaction method: For gesture-based interaction, a hand appears that repeats the correct gesture until the gem is successfully selected. In voice-based interaction, the text “say <Select>” appears when a gemstone is targeted with the gaze cursor. However, the tutorial behaves the same way for an incorrect input (e.g., an incorrect gesture or voice command) and there is no feedback reporting that the input is incorrect. Therefore, if the child had a difficulty in understanding the instruction, the experimental supervisor helped by repeating or explaining the instruction given via voice and text. A successful selection is visually indicated by the vanishing of the selected gemstone and a short audio signal. Once all three gemstones are successfully selected, the tutorial automatically proceeds to the next task. To assess the efficiency of the different AR-interaction modes, we asked the children to perform each interaction mode for selecting a gemstone three times. We counted the number of attempts (to calculate the variable ‘mean number of attempts’) and the time (to calculate the variable ‘mean time’) for each of the three tries for ‘tap’, ‘air-tap’ and ‘voice command’. The tutorial performance was recorded with an external camera and with the POV-camera from the HoloLens 2 (see Figure 3) to validate the data for the two variables and to document possible technical issues that may influence the usability. After the tutorial, we asked the children to express their current activity-related achievement emotions with another questionnaire. They had to state their enjoyment, boredom and frustration in comparison to the previous questioning (variables ‘enjoyment-post’, ‘frustration-post, ‘boredom-post’) (see Document S1b). Pilot studies in which the questionnaires were developed had shown that children were very joyfully excited when they came to the experiment and therefore rated positive emotions as high as possible on the scale and negative emotions as low as possible on the scales. Adjustments were needed to allow the children to express that their positive emotions were even more positive or negative emotions were even less than before interacting with the AR-device. In addition, the children rated the overall system usability (variable ‘system usability score’) of the HoloLens 2 as an HMD-AR-device. The used System Usability Scale (SUS) [53] comprises ten statements on different facets of system usability. The statements were translated into German and the wording was simplified to match the target group of children (see Appendix B and Document S1c). To assess the children’s preference concerning the interaction modes provided by the device, we showed them schematic pictures of the ‘tap’-, ‘air-tap’- and ‘voice command’-interactions and re-explained the interaction modes to them after completing the tutorial. The children were asked to rank the interaction modes based on their personal preference (variables ‘most favorite interaction mode’ and ‘least favorite interaction mode’) and to provide explanations for their decisions. Finally, we asked the children to report any perceived inconveniences, e.g., pain from wearing the device or problems seeing objects or reading text in AR and to report any inconveniences that are related to simulator sickness [54].

### 2.4. Data Analysis

First, we calculated the SU score. Subsequently, we compared the interaction modes with respect to their efficiency and evaluated the children’s interaction preferences in AR. Lastly, we examined the changes in the activity-related achievement emotions.

#### 2.4.1. Overall Device Usability

The overall system usability was assessed using the variable ‘system usability score’. It is calculated as a descriptive measure from the children’s answers to the SUS-questionnaire. We used a five-point instead of the original ten-point-Likert scale to ease the rating process for the children and adjusted the score calculation accordingly. The scale is acceptably reliable (Cronbach’s Alpha = 0.76) for the sample. In addition, technical issues taken from the recorded tutorial videos that may impact the usability when wearing or using the device are described.

#### 2.4.2. Efficiency of the AR-Interaction Modes

The dependent variables ‘mean number of attempts’ and ‘mean time’ are compared for the three measurement points ‘tap’, ‘air-tap’ and ‘voice command’ using two respective non-parametric Friedman-tests for differences among repeated measures.

#### 2.4.3. Interaction Preference in AR

The quantitative distributions of the children’s most and least favorite interaction mode in AR are reported. Reasons given for their decisions are presented.

#### 2.4.4. Changes in Activity-Related Achievement Emotions

Changes regarding the activity-related achievement emotions were assessed by three Wilcoxon-signed-rank-tests for paired samples comparing the pre and posttest variables ‘enjoyment’, ‘boredom’ and ‘frustration’. Each emotion item of the pretest is based on a five-point Likert-scale (options: ‘totally disagree’—‘rather disagree’—‘neither’—‘rather agree’—‘totally agree’). In the second questionnaire the children rated their current emotions in comparison to the previous measuring point (options: ‘much less’—‘a little less’—‘unchanged’—‘a little more’—‘much more’). Therefore, data transformation was applied. For further details see Appendix A.

## 3. Results

### 3.1. Overall Device Usability

The ‘system usability score’ was calculated based on the children’s answers on the five-point Likert-scale with high ratings on regular items increasing the score and high ratings on inverse items decreasing the score following Brooke [53] (see Document S1c). The median system usability score is 80 (maximum: 100) which indicates a ‘good’ system usability [55].

Apart from minor (situational) technical issues, one (that may impact the device usability) occurred frequently: In 19 out of 43 subjects (56%), the AR-objects moved away from the child’s hand as it approached the object. This occurred when the child had to move the body towards an object to reach it, but not when the child stood still when interacting with an object. For a demonstration of the issue see Video S1d. Furthermore, none of the subjects reported any physical inconveniences related to simulator sickness.

### 3.2. Efficiency of the AR-Interaction Modes

The two non-parametric Friedman-tests of differences among repeated measures (for descriptive statistics see Table 1) revealed significant differences among the three interaction modes ‘tap’, ‘air-tap’ and ‘voice command’ concerning the required number of attempts (*Chi-Square* (2) = 72.29, *p* < 0.001) and the required time (*Chi-Square* (2) = 82.19, *p* < 0.001). Pairwise post-hoc comparisons (see Table 2) indicate that the ‘air-tap’-interaction requires more attempts compared to ‘tap’ and ‘voice command’. Concerning the required time, all three interaction modes differ significantly, with ‘tap’ being the fastest and ‘air-tap’ requiring the most time to perform. To conclude, the ‘tap’-interaction appears to be the most efficient for both interaction preferences. The ‘voice command’-interaction appears to be the second-best interaction mode. The ‘air-tap’-interaction is noticeably less efficient to use.

### 3.3. Interaction Preferences in AR

**Figure 4:**
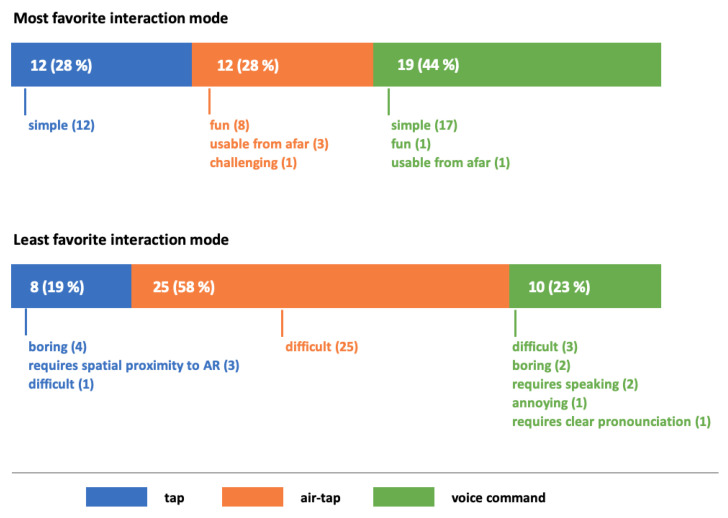
Distributions for most and least favorite interaction mode in AR with reasons for decisions (N = 43).

As shown in Figure 4, most children preferred the ‘voice command’-interaction. ‘Tap’ and ‘air-tap’ were chosen equally frequent. The most often stated reason for the choice of preference was the simplicity of interaction in AR (for ‘tap’ and ‘voice command’). The children who favored the ‘air-tap’-interaction mostly argued that it was fun to use. However, almost 60 percent of the children stated ‘air-tap’ as their least favorite interaction mode, arguing that it was difficult to use. ‘Tap’ and ‘voice command’ were chosen almost equally frequent for various reasons.

### 3.4. Changes in Acitivity-Related Achievement Emotions

The Wilcoxon-signed-rank-tests for paired samples (see Table 3) revealed an increase in enjoyment (*Z* = −5.641, *p* < 0.001), and a decrease in boredom (*Z* = −5.031, *p* < 0.001) and frustration (*Z* = −5.097, *p* < 0.001).

## 4. Discussion

To sum up, HoloLens 2 has proven to be an effective and appropriate HMD-AR-device to use with elementary school children, also confirmed by their subjective usability ratings. While children managed to use all provided interaction modes, the newly implemented direct ‘tap’-interaction turned out to be the best performing mode of interaction with virtual elements. Their reported most favorite interaction mode, however, was the ‘voice-command’-interaction. Although children were unfamiliar with HMD-AR-devices, the positive effects on activity-related achievement emotions suggest that educational settings may even benefit from using the HoloLens 2.

### 4.1. Overall Device Usability

The usability of the device was rated with an average system usability score of 80, resulting in the rating ‘good’ (scores greater than 82 indicate “excellent” usability) [55]. Still, further improvements are required for an effective application of the HoloLens 2 in educational applications because any usability flaw can hamper the learning performance (see Section 1.2). This can be achieved through, e.g., further technical improvements or specific tutorials adapted to the respective application and target group. As AR-technology and AR interactions were largely unknown to the children, it is possible that the extensive instruction required before and during the tutorial could have had a negative impact on the system usability score.

The frequently observed technical issue of AR objects moving away from children could stem from the device settings concerning the relative positioning of AR objects to the viewer, which seem to be designed for adults and their body dimensions. This did not occur in any of the adults that used the device. However, this is a preliminary statement without statistical validation as we did not investigate the occurrence of this issue comparing children with adults. It is assumed that the spatial position of the AR-objects in the tutorial is preset for adults and their physical appearance and that the children need to make a step towards an object to reach it. Yet, this causes the AR-objects to move away from the child as the device registers the spatial movement of the child and tries to maintain the relative position between spectator and AR-object. For a successful use of the device in educational situations with young children, the relative positioning of AR-objects should therefore either be scaled down to the length of children’s arms or absolute object positioning should be selected instead.

The absence of physical inconveniences related to simulator sickness might be explained by the fact that the familiar real world remains visible in AR. Our results are consistent with findings for the HoloLens (first generation) [56], that revealed the device to only cause negligible symptoms of simulator sickness in training. Simulator sickness has so far been observed more frequently in VR experiences [57], in which the subjects are visually isolated from their reference world.

### 4.2. Efficiency of the AR-Interaction Modes

As described in Section 3.2, we found the ‘tap’-interaction to be most efficient concerning our applied measures (mean task attempts, mean task time) without prior training for the children, as it requires a low number of attempts and can be performed quickly. The ‘voice command’-interaction was found to be the second-best interaction mode (low number of attempts, but higher required performance time). The ‘air-tap’ mode is the least efficient to use, as it requires both a high number of attempts and a long time to perform. Based on our experience during the data collection, this might be since the ‘air-tap’-interaction is only registered successfully by the device if the object is selected by the hand beam long enough and if the ‘tap’ gesture is performed clearly. It is possible that the children’s physical and motoric body characteristics [42] additionally complicated the detection of the gesture.

As we found the mean task time to be significantly higher for ‘voice command’ in comparison to ‘tap’, which we assume is caused by the longer action processing time of the voice-and-gaze-based interaction. It is necessary for the person to say the command, and for the device to detect and process it, while for the gesture-based interactions, the device can make use of the real-time gesture tracking. In their study with the predecessor model (HoloLens first generation), Munsinger et al. [43] found the ‘voice command’-interaction to produce more input errors than the ‘air-tap’-interaction. They further found the ‘voice command’ to require more time to perform than the ‘clicker’ (which is not available for the HoloLens 2), but not more time than the ‘air-tap’-interaction. Our results deviate from these findings as we found the ‘voice command’-interaction of the latest HoloLens to be noticeably improved and generally efficient and to not be overall inferior to the best performing interaction mode (i.e., the direct ‘tap’-interaction, which was not available on the HoloLens first generation). This improvement was evident even though the children in this study wore medical face masks, which may have limited the clarity of their pronunciation. However, we cannot accurately compare our results concerning the pairwise comparisons between the AR-interaction mode, as we used different study designs (within-subjects design vs. experimental design) and different procedures (focus on the first three attempts with an interaction mode vs. observation after a familiarizing phase).

In our model, we assume that a higher required number of attempts indicates a lower efficiency. However, especially for gesture-based AR-interaction, it could be more efficient to perform several attempts in a short period of time than to only make one attempt. On the other hand, the opposite might be the case for voice-and-gaze-based interaction in AR, based on our experience. Consequently, we did not compute a single efficiency measure from the two measures used in this study, as they could scale in different directions in terms of efficiency. Therefore, the interpretation of the calculated measures regarding the efficiency of the methods must be done cautiously. The efficiency effect of performing several gesture-attempts in a shorter period appeared to be evident for the ‘air-tap’-interaction, which is the more complex gesture-based AR-interaction mode in this study. This effect might even be more prominent in more complex gesture-based interactions like the rotation of an object. However, the measure ‘mean time’ appears to be overall applicable for interpretation concerning efficiency, as a lower completion time means higher efficiency for any AR-interaction mode.

### 4.3. Interaction Preferences in AR

We found that the children’s favorite mode to interact in AR was the ‘voice command’. However, ‘tap’ and ‘air-tap’ were chosen equally often. We therefore conclude that the preferences concerning AR-interaction vary among the children. For ‘tap’ and ‘voice command’, the most given reason for the stated preference was the perceived simplicity of the task. This indicates that the children made their choice based on their personal experience with the respective AR-interaction modes. Yet, it appears that they did not necessarily make their choice based on the efficiency measures that we applied to compare the AR-interaction modes since the ‘voice-command’-interaction was chosen most often It could be the case that the children who favored the ‘voice-command’-interaction found it easier to say the command than to tap at the object, despite performing ‘more efficient’ with the ‘tap’-interaction. The children that favored the ‘air-tap’-interaction mostly argued that it was fun to use. It appears that this choice was not made based on efficiency, but rather on personal enjoyment. However, the ‘air-tap’-interaction appears to be the least favored in general as almost 60 percent of the children claimed to not like it because of its difficulty to perform. This is consistent with our conclusion that it is the least efficient mean to interact in AR.

### 4.4. Activity-Related Achievement Emotions

With the aim of evaluating the applicability of the HoloLens 2 for a target group of children in educational settings, we also analyzed the influence of the HMD-AR-device on activity- related achievement emotions. Results are consistent with prior research [31] and indicate a positive effect of the AR-device on enjoyment, which is assumed to promote motivation and interest [32,33]. Although all children used the innovative and technically challenging AR-device for the first time, results showed a decrease in frustration and boredom, being another hint for its motivational effects. While these promising results on emotions point at possible learning benefits for HMD-AR-supported learning environments, it remains unclear whether the effects persist over time as the children become used to the device and the AR-experience. Moreover, it cannot be assumed that positive emotions concerning learning materials or specific devices improve learning outcomes per see [58]. According to the Cognitive Affective Theory of Multimedia Learning (*CATML* [59]), emotions impact cognitive processes during multimedia learning, as they affect the learners’ engagement. The processes involved are complex. It has been demonstrated, for example, that too much positive emotion can impede learning, and that in some cases even negative emotions can be useful [60]. Thus, when devices that trigger strong emotions are used for learning, it is important to carefully examine what effect this has on learning. Therefore, future studies should address affective processes in learning with the HoloLens 2.

### 4.5. General Limitations of the Study

Due to the necessity to make individual appointments in a laboratory at a university, the sample could have a high proportion of children who are rather interested in AR (or technology in general) as they participated in the study on a voluntary basis and were not invited as a school class. The likely very high proportion of children in the sample with a strong interest in AR could reduce the transferability of the results to the overall population. Therefore, further research with other samples is generally needed to validate or complement the results.

Moreover, the order of the AR-interaction mode presentation in the tutorial was not randomized. The lack of randomization may have produced carry-over effects from the first measuring point (‘tap’) to the second (‘air-tap’) and to the third (‘voice command’). However, this carry-over could have been diminished by the difference in execution between the presented AR-interaction modes, thus not offering many possibilities to learn something from a task that could be used when performing the next. In an experimental design with split groups for each interaction mode however, we would not have been able to assess the children’s personal interaction preference in AR in such semantic proximity to the actual tasks that they experienced as in the within-design that we chose.

Due to the lack of an underlying learning content in the study, the findings cannot be directly transferred into educational situations, and they should be validated or complemented in further studies using multiple activities and learning contents as examples. Nevertheless, the present results can serve as a general basis for the development of any educational HMD-AR application.

Concerning the lack of technical adaption of the device to the physical body characteristics of young children (especially the lower interpupillary distance), we found that although the device was not purposely developed for children, it proved to be usable. In our study, none of the children reported problems seeing virtual objects (neither when asked right after the eye calibration at the start of the study nor after the tutorial when we asked them to report symptoms related to simulator sickness, including problems with text reading or image perception in AR).

### 4.6. Practical Application

Research findings pointing to the general benefits of AR in educational contexts, and the present study demonstrating that HMD-AR has the potential to be used with younger learners, suggest that in the future, HMD-AR could be profitably used in classrooms. However, from an economic point of view, smart glasses are very expensive devices and if they are purchased in large numbers, e.g., for a complete course, they represent a high financial outlay for schools. In addition, there may be high software development costs, which are necessary because there are still hardly any purchasable software products for curricular-relevant contents. Therefore, schools and other educational institutions are well advised to implement AR in the classroom only if it has been proven that a specific learning content is better conveyed through AR than via traditional or less expensive media and methods. For learning content in which manual interaction with real or virtual objects is not central to learning success, one can also fall back on the more affordable tablet variant.

## 5. Conclusions

Our study indicates a good system usability of Microsoft’s HoloLens 2 when used by elementary school children. The newly implemented direct ‘tap’-interaction in AR appears to be most effective without prior training. Despite requiring more time to perform, the ‘voice command’-interaction was found to work well with children’s voices. This deviates from previous findings for the HoloLens (first generation) and other technologies. Further, we found different interaction preferences in AR among the children in accordance with prior research. Yet, the children’s preferences do not seem to be based on objective efficiency. Our study suggests that the HoloLens 2 (as an HMD-AR-device) is generally effective for applications with young children as a target group. However, the provided AR-interaction modes appear to differ in their efficiency, at least during the time of familiarization with the device. We propose that future HMD-AR applications for education offer multiple interaction modes to serve the different interaction preferences in HMD among the children.

Although the research results presented are not based on a specific learning content, they still provide an important point of reference for developers designing HMD-AR based learning applications. However, the particular integration of HMD-AR in educational situations has to be aligned with applied instructional methods and current learning goals. Prior research on HMD-VR-based lessons showed that learning outcomes vary depending on the use of a learning strategy [61]. Nevertheless, the detachment from a learning content allows our findings to be used as a basis to determine general requirements, possibilities, and limitations of the development and implementation of any kind of educational HMD-AR-environments for children. Although our study suggests that the device can be successfully used with elementary school children, a technical adaption concerning the physical body characteristics (e.g., the adaption to lower interpupillary distance) needs to be carried out. Furthermore, more research is needed to verify and complement our findings, as the evaluated device and its associated technologies are still a novelty, with little directly related research available in general. However, due to the continuous technical development of HMD-AR devices, our research only describes the current status and must be revised with the appearance of a successor model or another, better device. Future research in this area could assess the usability of the device in educational settings and compare the usability of the provided AR-interaction modes for actions of higher complexity, e.g., rotating an AR-object or altering its size.

## Figures and Tables

**Table 1:** Descriptive statistics for the efficiency assessment.

Dependent Variable	Mean (SD)
mean number of attempts for ‘tap’	1.001 (0.508)
mean number of attempts for ‘air-tap’	2.763 (1.549)
mean number of attempts for ‘voice command’	1.194 (0.771)
mean time [s] for ‘tap’	1.200 (0.346)
mean time [s] for ‘air-tap’	16.047 (13.443)
mean time [s] for ‘voice command’	3.672 (6.007)

**Table 2:** Pairwise Dunn-Bonferroni post-hoc comparisons for the efficiency assessment.

Dependent Variable	Compared Interaction Modes	*Z*	*p* (Two-Tailed)	*r* (Cohen)
mean number of attempts	tap—air-tap	–1.49	<0.001 ***	0.227
	tap—voice command	0.22	0.306	
	air-tap—voice command	1.27	<0.001 ***	0.193
mean time	tap—air-tap	–1.95	<0.001 ***	0.298
	tap—voice command	–1.047	<0.001 ***	0.215
	air-tap—voice command	0.907	<0.001 ***	0.138

Significance levels: *** *p* < 0.001.

**Table 3:** Descriptive statistics and signs (Wilcoxon-signed-rank-test) for the for the activity emotion change assessment.

Dependent Variable	Mean ^1^ (SD)	Pos. Signs	Neg. Signs	Ties
enjoyment-pre	6.350 (0.613)	36	0	7
enjoyment-post	7.880 (0.981)			
boredom-pre	3.370 (0.817)	2	30	11
boredom-post	2.090 (1.250)			
frustration-pre	3.050 (0.213)	1	27	15
frustration-post	1.840 (1.022)			

^1^ Range for transformed means: 1(very low)—9(very high).

**Figure A1:**
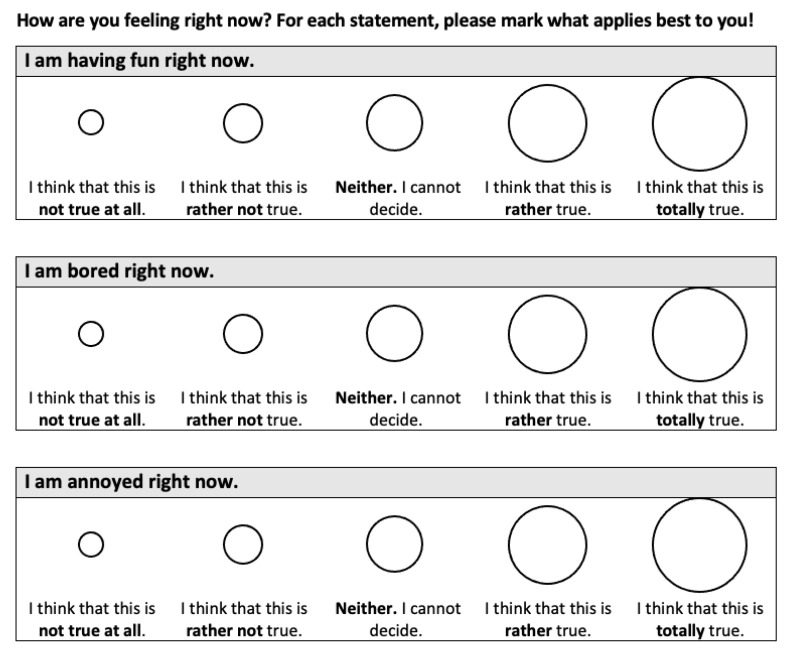
Pre-questionnaire for activity-related achievement emotions assessment.

**Figure A2:**
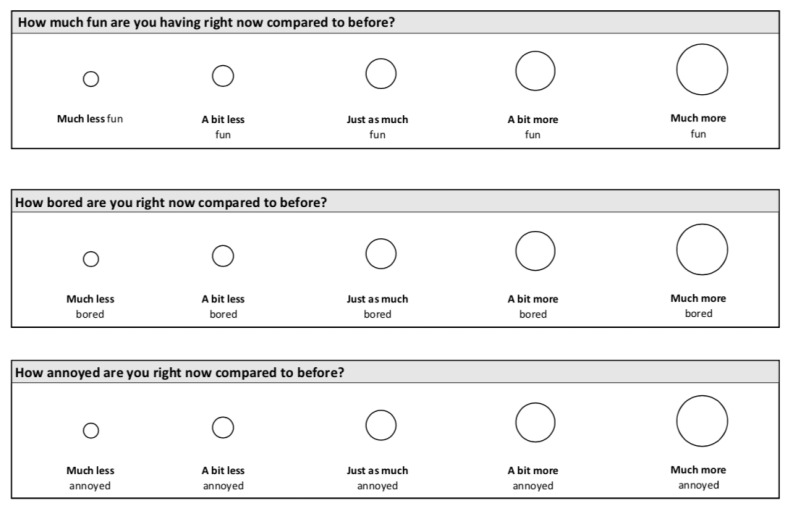
Post-questionnaire for activity-related achievement emotions assessment.

**Figure A3:**
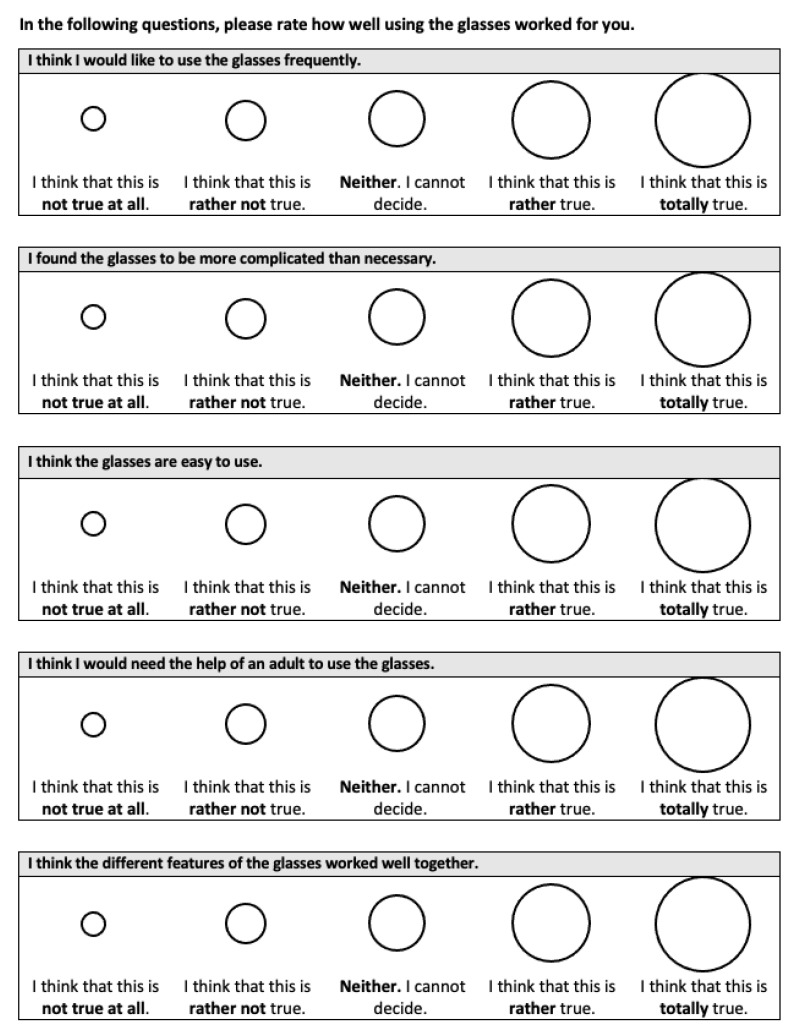
Questionnaire for device usability assessment (page 1).

**Figure A4:**
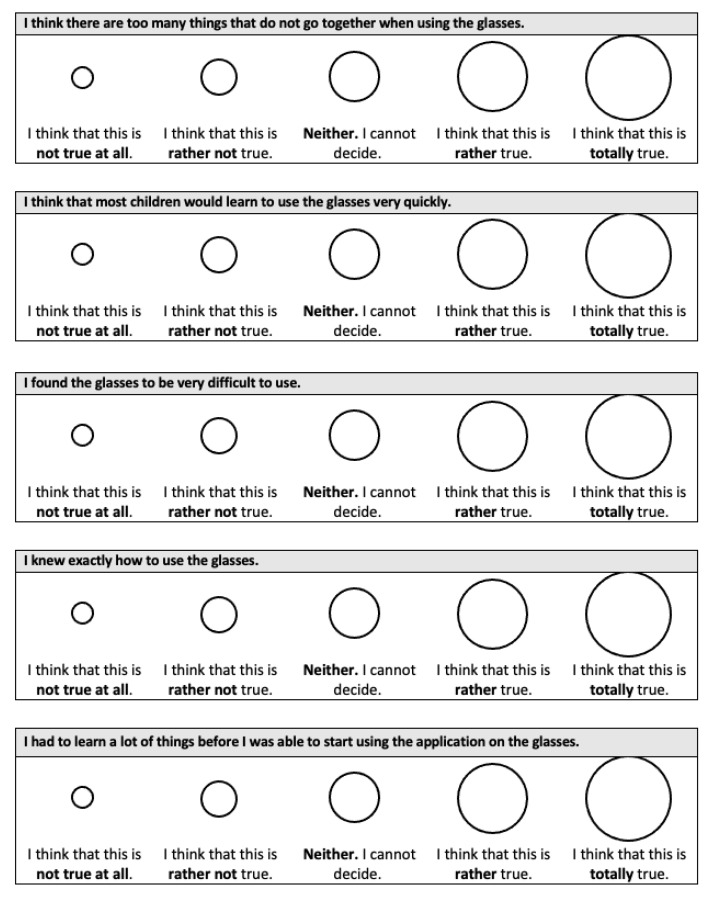
Questionnaire for device usability assessment (page 2).

## Data Availability

The data presented in this study are openly available in Open Science Framework (OSF) via: https://mfr.osf.io/render?url=https%3A%2F%2Fosf.io%2Fdf6w9%2Fdownload (31 August 2021). For reasons of data protection, the video recordings are not publicly accessible.

## Supplementary Materials

The following will be made available online at https://www.mdpi.com/article/10.3390/s21196623/s1, Video S1a: tutorial demo with selected samples, Document S1b: activity emotion questionnaire, Document S1c: system usability questionnaire, Video S1d: spatial repositioning issue demonstration.

## Appendix A

The children’s activity-related achievement emotions were assessed before and after the tutorial on interaction in HMD-AR using the pre-questionnaire comprises three items adapted for children from Riemer and Schrader [52] (see Figure A1). As explained in Section 2.3., pre-studies for the development of the questionnaires had shown that the children rated positive emotions as high as possible on the scale and negative emotions as low as possible on the questionnaire before and after the tutorial. We therefore modified the post-questionnaire to allow children to express their emotions in relation to the previous questioning (see Figure A2).

As described in Section 2.4.4, data transformation was required for the activity emotion assessment. Both questionnaires are based on a five-point Likert-scale, but the second questionnaire asks the children to rate their sentiments in comparison to the previous questioning. To take this comparison into consideration, we transformed the dataset from the first questionnaire from a range of 1–5 to 3–7, and for the second questionnaire, we either increased or reduced the previous value based on the chosen answer on the items as shown in Table A1.

**Table A1:** Data transformation for activity-related achievement emotions assessment.

**Pre-test item, e.g., ‘I am having fun right now.’**					
**Answer**	**‘Totally disagree’**	**‘Rather disagree’**	**‘Neither’**	**‘Rather agree’**	**‘Totally agree’**
**Pre-Score X**	3	4	5	6	7
**Post-test item, e.g., ‘How much fun you are having right now in comparison to before?’**					
**Answer**	‘Much less’	‘A little less’	‘Unchanged’	‘A little more’	‘Much more’
**Post-Score Y**	X − 2	X − 1	X	X + 1	X + 2

## Appendix B

The questionnaire for the overall device usability assessment described in Section 2.3. consists of the ten items of the System Usability Scale (SUS) [53] (see Figure A3 and Figure A4). The items were translated to German and the wording was simplified.
