# Supplementary material for: Investigating the Usability of a Head-Mounted Display Augmented Reality Device in Elementary School Children
Source: Sensors (Basel). 2021 Oct 5;21(19):6623. doi: 10.3390/s21196623 (PMC8512836; doi:10.3390/s21196623)
Supplement: Supplementary file 1 [file sensors-21-06623-s001.zip › sensors-1383925-supplementary/SuppFiles/Document_S1b_activity_emotions_questionnaire .pdf]

How are you feeling right now? For each statement, please mark what applies best to you!

**I am having fun right now.**				
○	○	○	○	○
I think that this is **not true at all**.	I think that this is **rather not** true.	**Neither.** I cannot decide.	I think that this is **rather** true.	I think that this is **totally** true.

**I am bored right now.**				
○	○	○	○	○
I think that this is **not true at all**.	I think that this is **rather not** true.	**Neither.** I cannot decide.	I think that this is **rather** true.	I think that this is **totally** true.

**I am annoyed right now.**				
○	○	○	○	○
I think that this is **not true at all**.	I think that this is **rather not** true.	**Neither.** I cannot decide.	I think that this is **rather** true.	I think that this is **totally** true.

How are you feeling right now? For each statement, please mark what applies best to you!

How much fun are you having right now compared to before?

○	○	○	○	○
**Much less** fun	**A bit less** fun	**Just as much** fun	**A bit more** fun	**Much more** fun

How bored are you right now compared to before?

○	○	○	○	○
**Much less** bored	**A bit less** bored	**Just as much** bored	**A bit more** bored	**Much more** bored

How annoyed are you right now compared to before?

○	○	○	○	○
**Much less** annoyed	**A bit less** annoyed	**Just as much** annoyed	**A bit more** annoyed	**Much more** annoyed
