# Supplementary material for: Investigating the Usability of a Head-Mounted Display Augmented Reality Device in Elementary School Children
Source: Sensors (Basel). 2021 Oct 5;21(19):6623. doi: 10.3390/s21196623 (PMC8512836; doi:10.3390/s21196623)
Supplement: Supplementary file 1 [file sensors-21-06623-s001.zip › sensors-1383925-supplementary/SuppFiles/Document_S1c_usability_questionnaire.pdf]

In the following questions, please rate how well using the glasses worked for you.

I think I would like to use the glasses frequently.

I think that this is **not true at all**.	I think that this is **rather not** true.	**Neither**. I cannot decide.	I think that this is **rather** true.	I think that this is **totally** true.

I found the glasses to be more complicated than necessary.

I think that this is **not true at all**.	I think that this is **rather not** true.	**Neither.** I cannot decide.	I think that this is **rather** true.	I think that this is **totally** true.

I think the glasses are easy to use.

I think that this is **not true at all.**	I think that this is **rather not** true.	**Neither.** I cannot decide.	I think that this is **rather** true.	I think that this is **totally** true.

I think I would need the help of an adult to use the glasses.

I think that this is **not true at all.**	I think that this is **rather not** true.	**Neither.** I cannot decide.	I think that this is **rather** true.	I think that this is **totally** true.

I think the different features of the glasses worked well together.

I think that this is **not true at all**.	I think that this is **rather not** true.	**Neither.** I cannot decide.	I think that this is **rather** true.	I think that this is **totally** true.

I think there are too many things that do not go together when using the glasses.

I think that this is **not true at all**.	I think that this is **rather not** true.	**Neither.** I cannot decide.	I think that this is **rather** true.	I think that this is **totally** true.

**I think that most children would learn to use the glasses very quickly.**				
I think that this is **not true at all**.	I think that this is **rather not** true.	**Neither.** I cannot decide.	I think that this is **rather** true.	I think that this is **totally** true.

**I found the glasses to be very difficult to use.**				
I think that this is **not true at all**.	I think that this is **rather not** true.	**Neither.** I cannot decide.	I think that this is **rather** true.	I think that this is **totally** true.

**I knew exactly how to use the glasses.**				
I think that this is **not true at all**.	I think that this is **rather not** true.	**Neither.** I cannot decide.	I think that this is **rather** true.	I think that this is **totally** true.

**I had to learn a lot of things before I was able to start using the application on the glasses.**				
I think that this is **not true at all**.	I think that this is **rather not** true.	**Neither.** I cannot decide.	I think that this is **rather** true.	I think that this is **totally** true.
